# Supplementary material for: Activation and allosteric regulation of the orphan GPR88-Gi1 signaling complex
Source: Nat Commun. 2022 May 2;13:2375. doi: 10.1038/s41467-022-30081-5 (PMC9061749; doi:10.1038/s41467-022-30081-5)
Supplement: Supplementary file 1 — Supplementary Information [file 41467_2022_30081_MOESM1_ESM.pdf]

Supplementary Information for Activation and allosteric regulation of the orphan GPR88-Gi1 signaling complex

Chen *et al.*

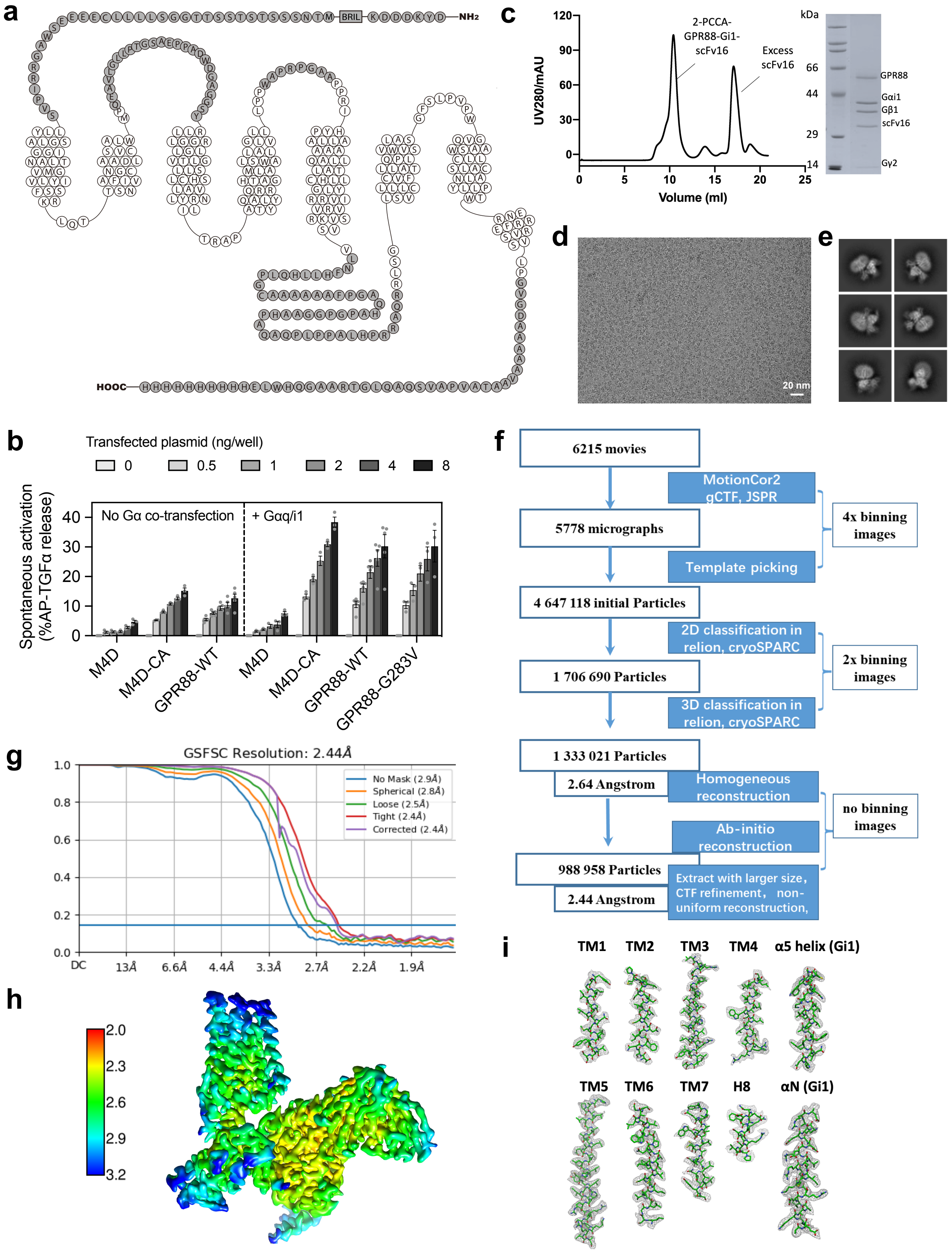

a
BRIL
NH2
HOOC
b
Transfected plasmid (ng/well)
0
0.5
1
2
4
8
No Gα co-transfection
+ Gαq/i1
Spontaneous activation (%AP-TGFα release)
M4D
M4D-CA
GPR88-WT
GPR88-G283V
c
UV280/mAU
Volume (ml)
2-PCCA-GPR88-Gi1-scFv16
Excess scFv16
kDa
GPR88
Gαi1
Gβ1
scFv16
Gγ2
d
20 nm
e
f
6215 movies
MotionCor2 gCTF, JSPR
5778 micrographs
Template picking
4x binning images
4 647 118 initial Particles
2D classification in relion, cryoSPARC
1 706 690 Particles
3D classification in relion, cryoSPARC
2x binning images
1 333 021 Particles
2.64 Angstrom
Homogeneous reconstruction
Ab-initio reconstruction
no binning images
988 958 Particles
2.44 Angstrom
Extract with larger size, CTF refinement, non-uniform reconstruction,
g
GSFSC Resolution: 2.44Å
No Mask (2.9Å)
Spherical (2.8Å)
Loose (2.5Å)
Tight (2.4Å)
Corrected (2.4Å)
DC
13Å
6.6Å
4.4Å
3.3Å
2.7Å
2.2Å
1.9Å
h
i
TM1
TM2
TM3
TM4
α5 helix (Gi1)
TM5
TM6
TM7
H8
αN (Gi1)

Supplementary Fig. 1: Construct, function and cryo-EM data processing of 2-PCCA-GPR88-Gi1 signaling complex. a Snake plot of secondary structure and amino acid sequence of GPR88 construct, regions that are not resolved in the cryo-EM map are labeled in gray. **b** Constitutive activity of GPCRs measured by the TGF-α shedding assay. Titrated GPCR plasmid (volumes per well in a 96-well plate) was transfected together with the AP-TGFα reporter plasmid in the presence or absence of the chimeric Gαq/i1 plasmid. After 1-day incubation, exogenous GPCR-dependent (i.e., mock signal-subtracted) AP-TGFα response was quantified. M4D, Gi-coupled M4-DREADD, which does not respond to the endogenous ligand, acetylcholine[84]. CA, constitutively active mutant. Note that as compared with the absence of Gαq/i1 transfection, co-expression of the Gαq/i1 chimera enhanced M4D-CA and GPR88-WT signals and that, unlike the ligand-induced signal (Supplementary Fig. S4), GPR88-WT and G283V showed a similar response. Bars and error bars represent mean and s.e.m. of 3 independent experiments, each performed in quadruplicate. **c** Size exclusion chromatography profile and SDS-PAGE of the 2-PCCA-GPR88-Gi1 complex from more than three independent experiments. **d** Representative micrograph of the complex particles from 5778 micrographs. **e** Representative 2D averages. **f** Workflow for cryo-EM image processing. **g** Gold standard Fourier shell correlation (FSC) curve indicates overall nominal resolution at 2.4 Å using the FSC=0.143 criterion. **h** Local resolution map. **i** Representative density maps and models for TM1-7 and H8 of GPR88 as well as the N-terminal and C-terminal α helices of Gαi1 (αN and α5). Source data are provided as a Source Data file.

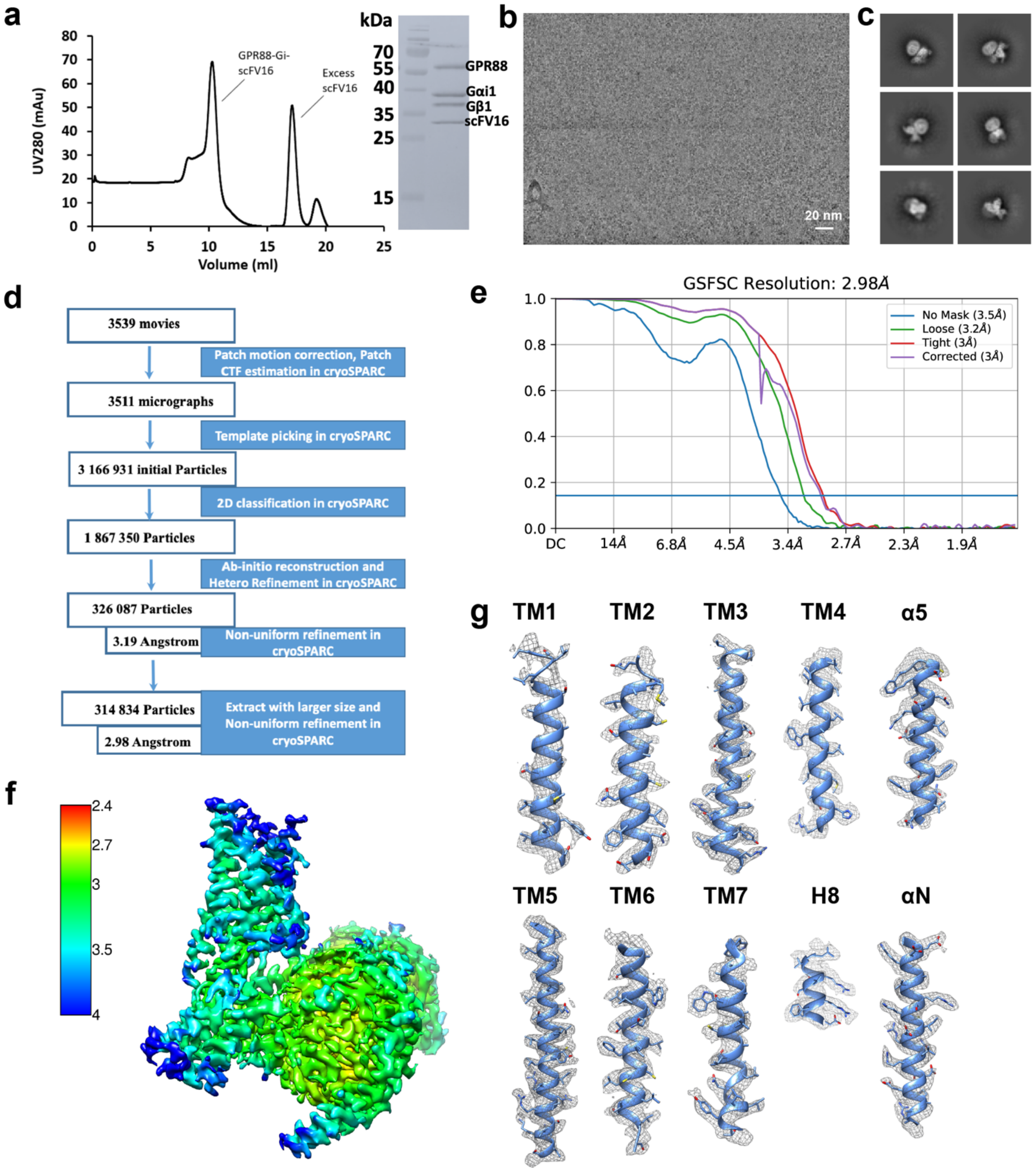

Supplementary Fig. 2: cryo-EM data processing of GPR88-Gi1 signaling complex without 2-PCCA. a Size exclusion chromatography profile and SDS-PAGE of the GPR88-Gi1 complex from more than three independent experiments. **b** Representative micrograph of the complex particles from 3511 micrographs. **c** Representative 2D averages. **d** Workflow

for cryo-EM image processing. **e** Gold standard Fourier shell correlation (FSC) curve indicates overall nominal resolution at 2.4 Å using the FSC=0.143 criterion. **f** Local resolution map. Source data are provided as a Source Data file. **g** Representative density maps and models for TM1-7 and H8 of GPR88 as well as the N-terminal and C-terminal α helices of Gαi1 (αN and α5).

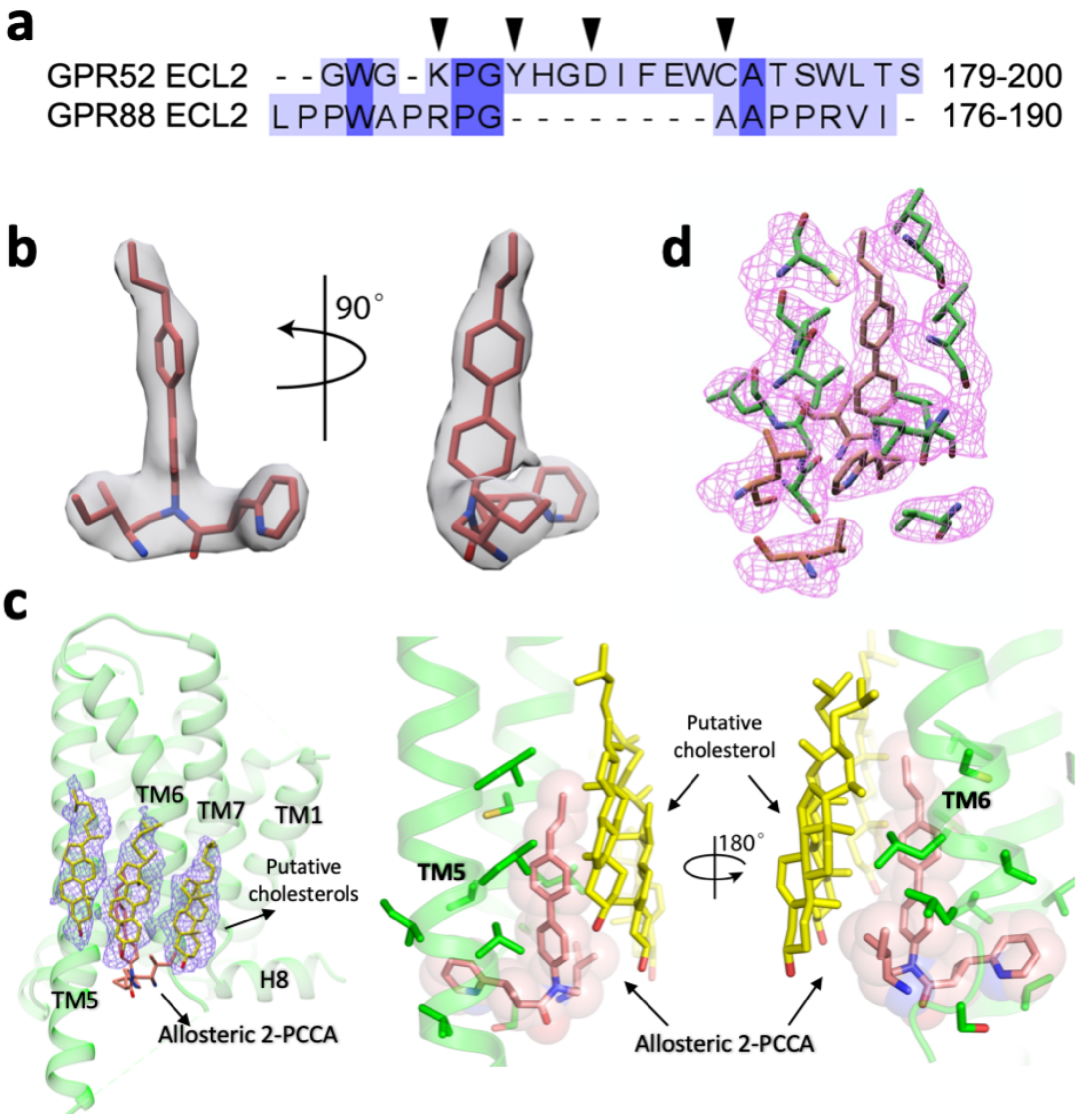

Supplementary Fig. 3: Sequence alignment and ligand electron density maps. a Structure-based sequence alignment of ECL2 between GPR52 and GPR88. **b** Density map of 2-PCCA from two different views. Black arrowheads indicate key residues for GPR52 self-activation. **c** Putative cholesterols observed on the side of the allosteric 2-PCCA. **d** Density map of residues of GPR88 that interact with 2-PCCA in the allosteric site.

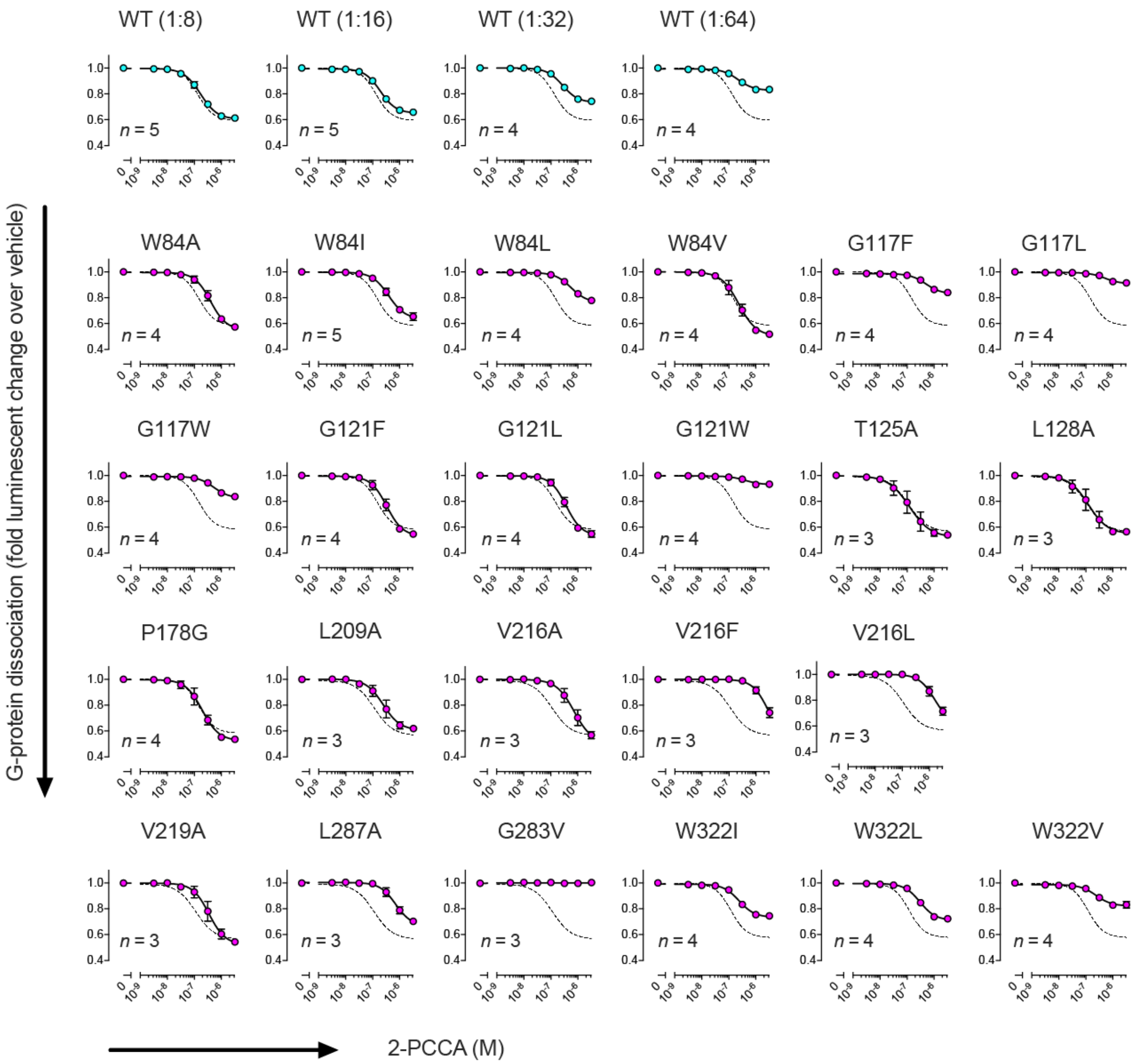

Supplementary Fig. 4: NanoBiT G-protein dissociation assay
Concentration–response curves for the NanoBiT-Gi dissociation signals for the titrated wild-type GPR88 and the indicated GPR88 mutants. Dashed line represents the wild-type GPR88 response. Symbols and error bars represent mean and s.e.m. of the indicated numbers of independent experiments, each performed in duplicate.

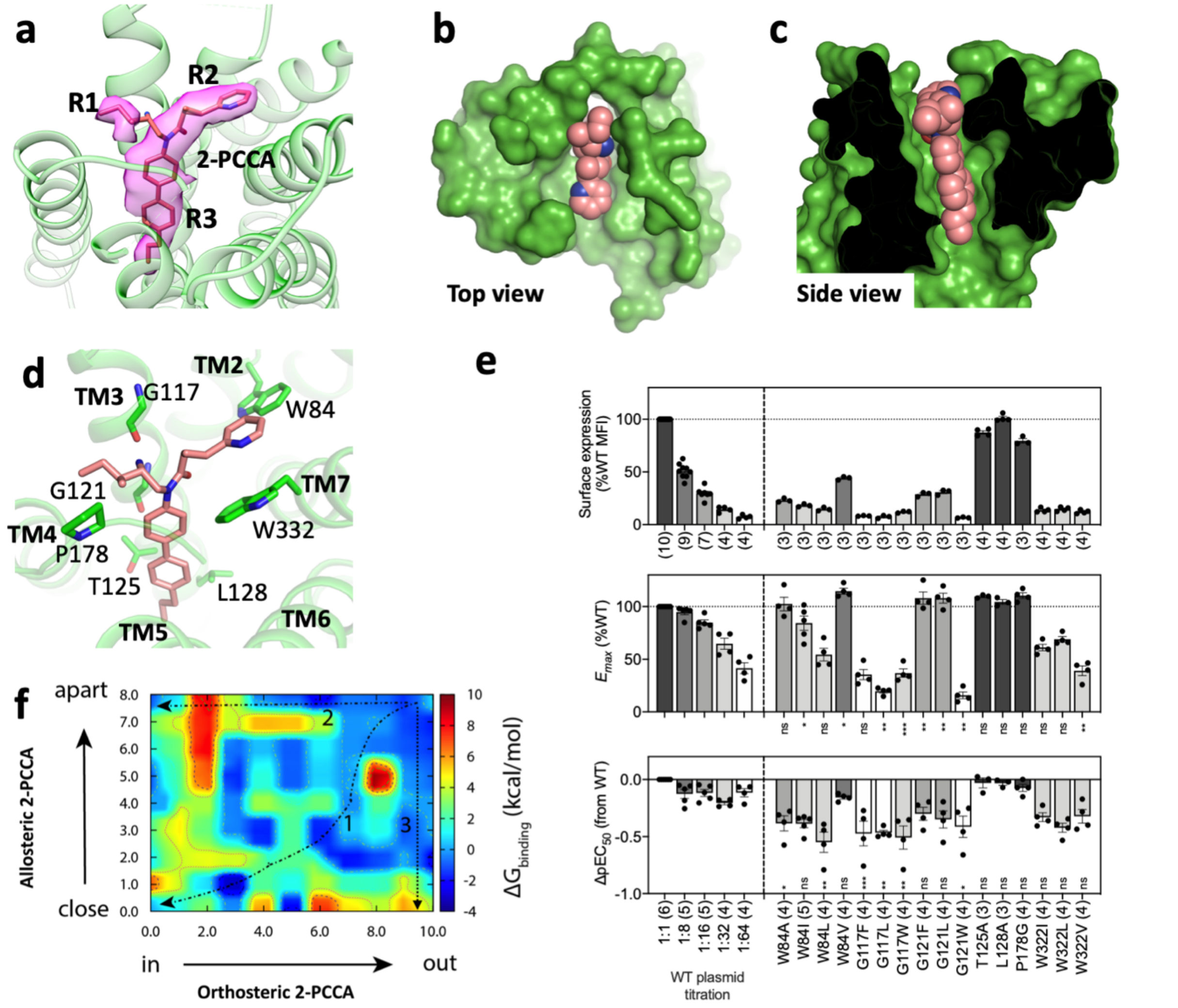

Supplementary Fig. 5: Potential interaction of 2-PCCA with the orthosteric site. a Modeling of 2-PCCA into the orthosteric density. **b, c** Molecular surface of the 2-PCCA-bound orthosteric pocket from top (**b**) and side (**c**) views. **d** Detailed interactions between 2-PCCA and the orthosteric pocket. **e** Cell surface expression and Gi-coupling activity were analyzed by the flow cytometry and the NanoBiT-Gi dissociation assay, respectively. From the concentration-response curves (Supplementary Fig. 4), E_{max} and ΔpEC_{50} values relative to the wild type were calculated. Colors in the mutant bars indicate an expression level matching to that of titrated wild type. Bars and error bars represent mean and s.e.m. of 3-6 independent experiments (dots), denoted as the parenthesis

at the bottom of the figure panels. NA, parameter not available because of lack of ligand response. Statistical analyses were performed using the ordinary one-way ANOVA followed by the two-sided Sidak's post-hoc test with the expression-matched (colored) WT response. ns, $p > 0.05$; *, $p < 0.05$; **, $p < 0.01$; ***, $p < 0.001$.**f** PDLD/s-LRA binding free energy surface of orthosteric and allosteric 2-PCCA molecules with respect to their geometry center distances (Ångstrom) away from experimental binding coordinates. Route 2 and 3 show the binding of a single ligand in the absence of the other has either high barrier or binding free energy. Coupling of the two binding processes lowers the barrier and validates synergic effect (route 1). Source data are provided as a Source Data file.

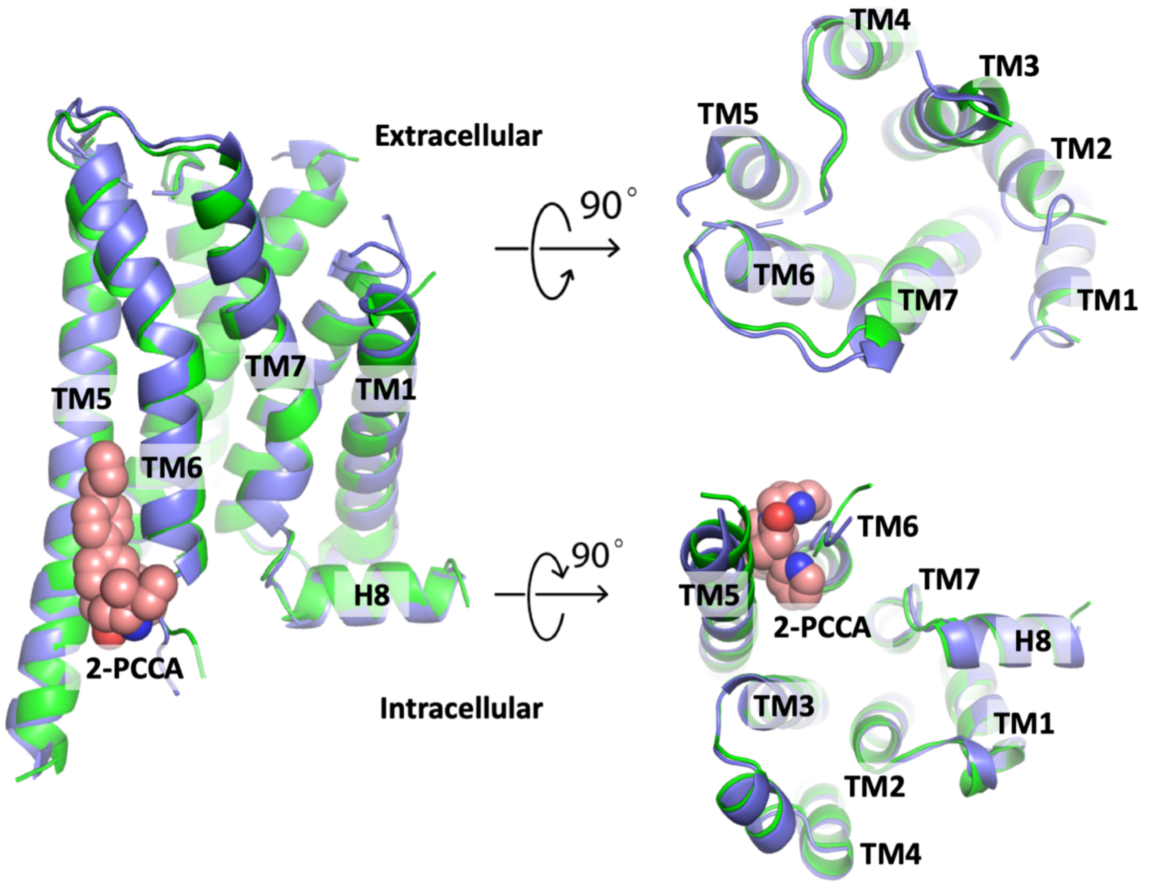

Supplementary Fig. 6: Structural superimposition of GPR88. Comparison of overall active GPR88 structures in the presence (green) or absence (blue) of 2-PCCA from the orthogonal, extracellular, and intracellular views. 2-PCCA is shown as sphere in salmon.

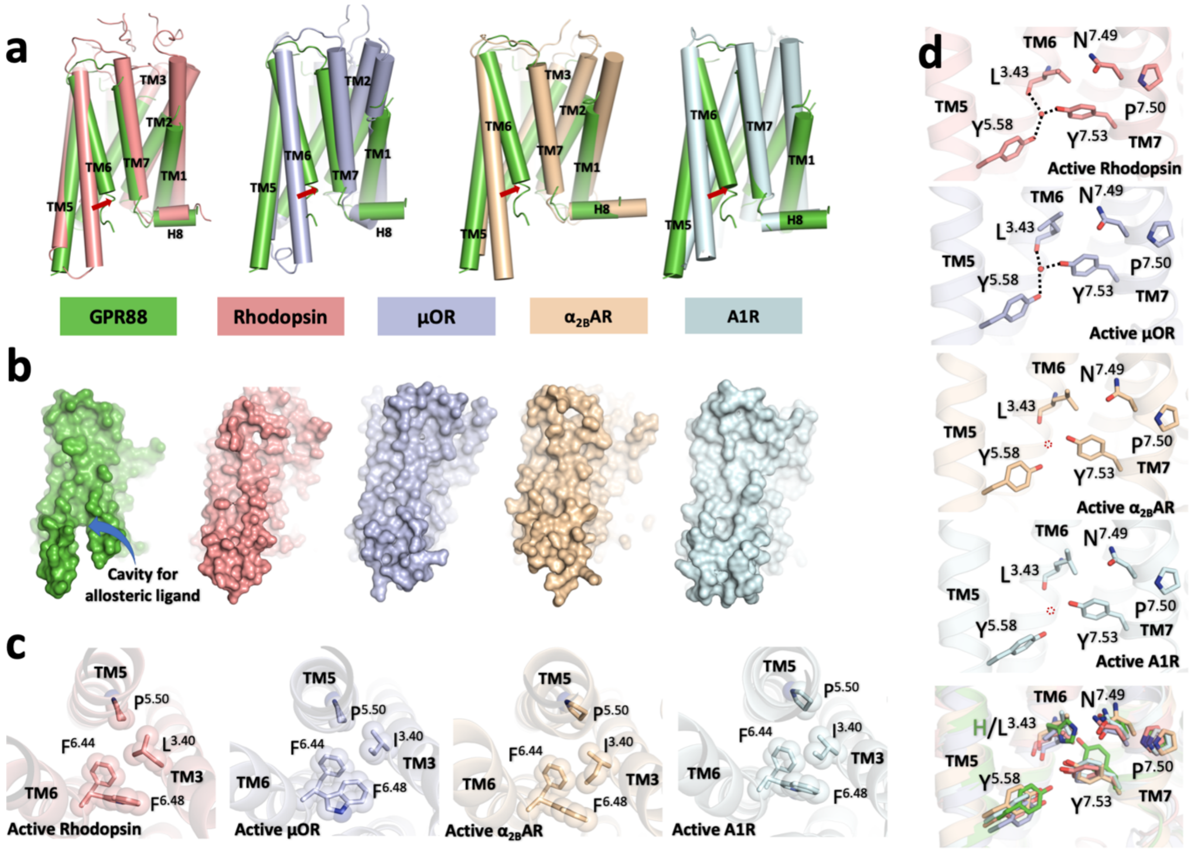

Supplementary Fig. 7: Comparison of the active structures of GPR88 and representative class A GPCRs. a Superimposition of active GPR88 structure with four different class A GPCRs rhodopsin (PDB:6CMO), μOR (PDB:6DDE), α_{2B}AR (PDB:6K42) and A1R (PDB:6D9H). **b** Comparison of the surfaces of the TM5 and TM6 interface for GPR88 and other GPCRs. **c** The active conformation of the conserved toggle switch $W^{6.48}$ and $P^{5.50}$-I/$L^{3.40}$-$F^{6.44}$ motif. **d** The water-mediated polar interactions between the conserved $Y^{5.58}$ and the $N^{7.49}P^{7.50}xxY^{7.53}$ motif.

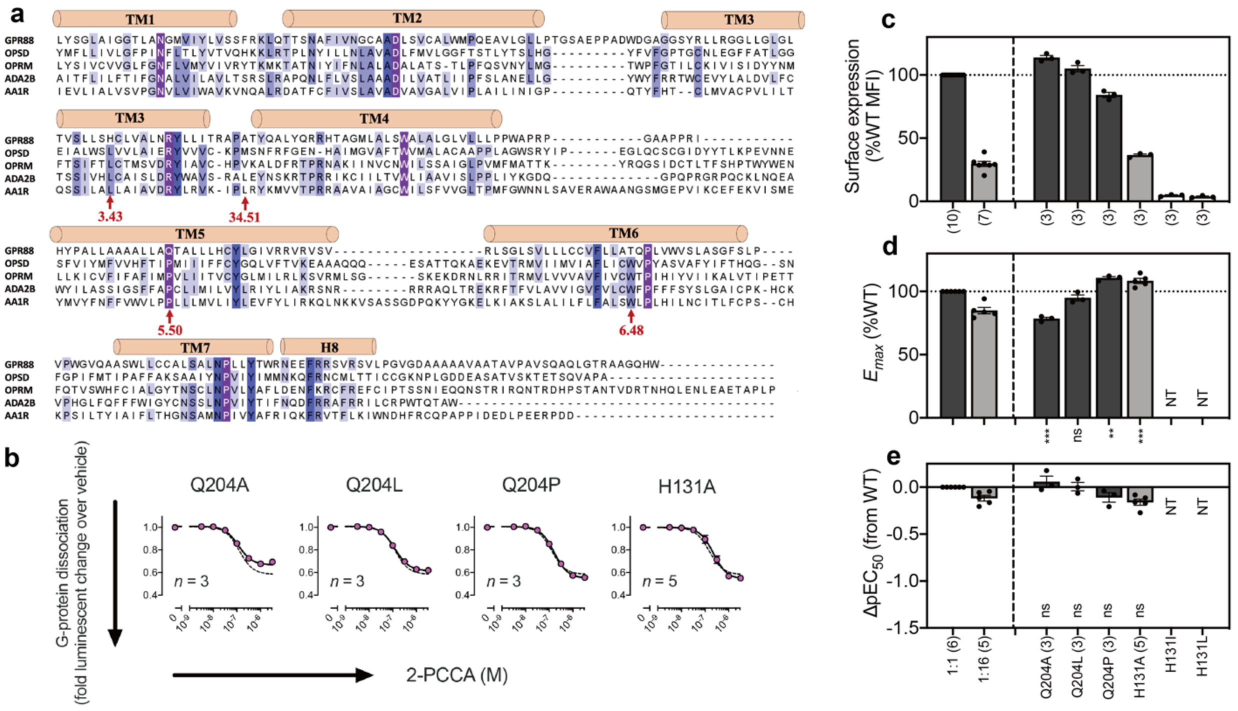

Supplementary Fig. 8: Sequence alignment and mutant study for $Q204^{5.50}$ and $H131^{3.43}$. **a** Structure-based sequence alignment of GPR88 with representative class A GPCRs, residues 3.43, 34.51, 5.50 and 6.48 are highlighted as red arrows. Mutant study for $Q204^{5.50}$ and $H131^{3.43}$. **b** Concentration–response curves for the NanoBiT-Gi dissociation signals. Dashed line represents the wild type GPR88 response (Fig. S1B). Symbols and error bars represent mean and s.e.m. of the indicated numbers of independent experiments, each performed in duplicate. **c** Cell surface expression analyzed by the flow cytometry. **d,e** Parameters for the Gi-coupling activity. From the concentration-response curves (**b**), Emax (**d**) and ΔpEC_{50} (**e**) values relative to the wild type were calculated. Colors in the mutant bars indicate an expression level matched to that of titrated wild type. Bars and error bars represent mean and s.e.m. of 3-6 independent experiments (denoted as the parenthesis at the bottom of the figure panels; note that d and e derived from the same dataset). NT, not tested because of too low surface expression levels. Statistical analyses were performed using the ordinary one-way ANOVA followed by the two-sided Sidak's post-hoc test with the expression-matched

(colored) WT response. ns, $p > 0.05$; *, $p < 0.05$; **, $p < 0.01$; ***, $p < 0.001$. Source data are provided as a Source Data file.

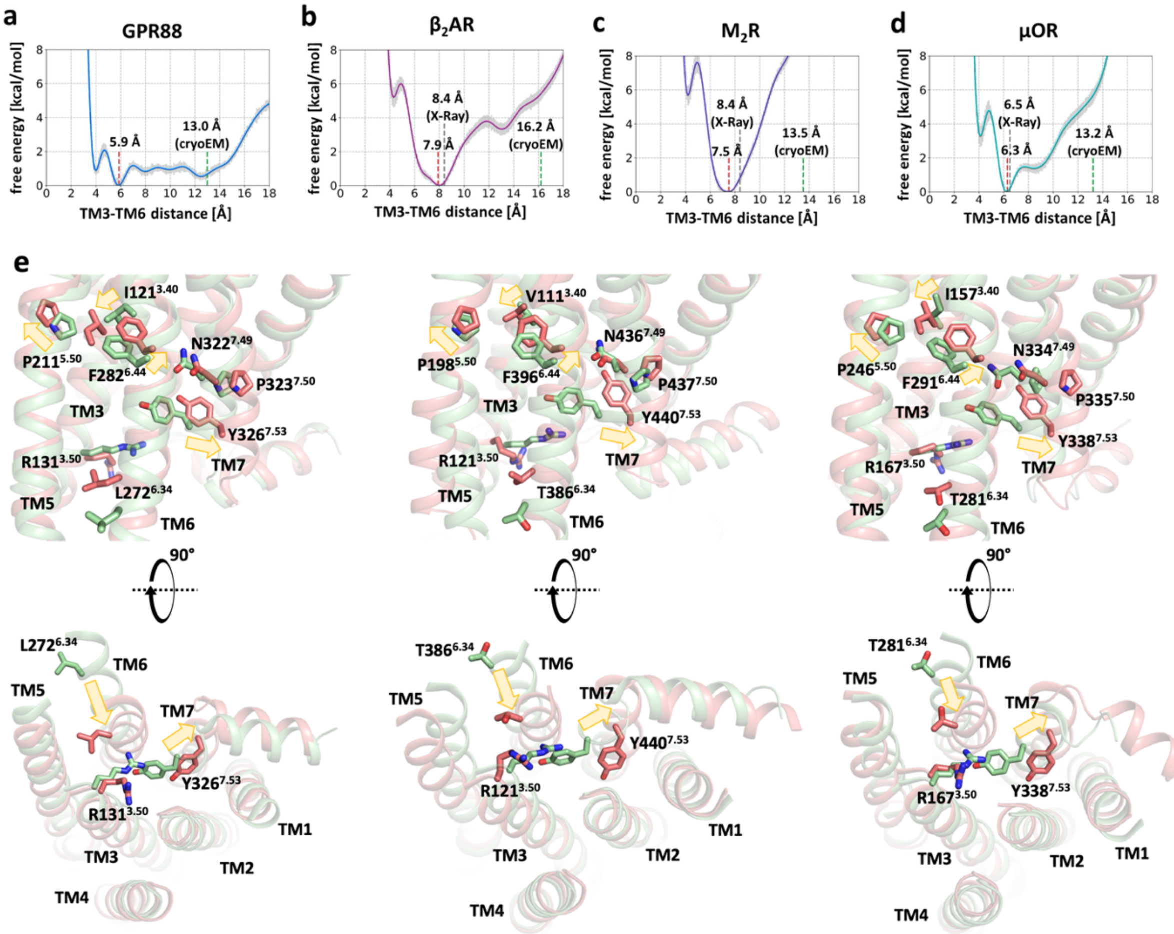

Supplementary Fig. 9: Deactivation protocol applied to GPR88, β2AR, M2R and μOR. a-d Free energy landscapes along the TM3-TM6 distance obtained by metadynamics simulations after 8.64 μs for GPR88 (**a**) and 10.56 μs for β2AR (**b**), M2R (**c**) and μOR (**d**). Error bars are indicated in gray and show that the global minima are more favorable compared to other local minima. **e** Calculated inactive models are shown in red and active-state cryo-EM structures in green (β2AR: PDB-ID 6NI3; M2R: PDB-ID 6OIK; μOR: PDB-ID 6DDE). Key residues comprising the $P^{5.50}$-$I^{3.40}$-$F^{6.44}$ / $P^{5.50}$-$V^{3.40}$-$F^{6.44}$ motif and $N^{7.49}P^{7.50}xxY^{7.53}$ motif as well as residues in position 3.50 and 6.34 are shown in sticks. Key conformational rearrangements are highlighted with yellow arrows. Data are presented as the reconstructed free energy average ± SEM in kcal/mol. Error analysis was performed applying block analysis

across the 32 walkers of each multiple walker metadynamics simulation (N=8.64×106 data points for GPR88 and N=10.56×106 data points for β2AR, M2R and μOR, each), following the protocol of the Plumed Master ISDD tutorial 2020 (https://www.plumed.org/doc-v2.6/user-doc/html/master-_i_s_d_d-2.html). A block size of 500 was applied.PDB files of the calculated inactive receptor models are provided as supplementary file. Source data are provided as a Source Data file.

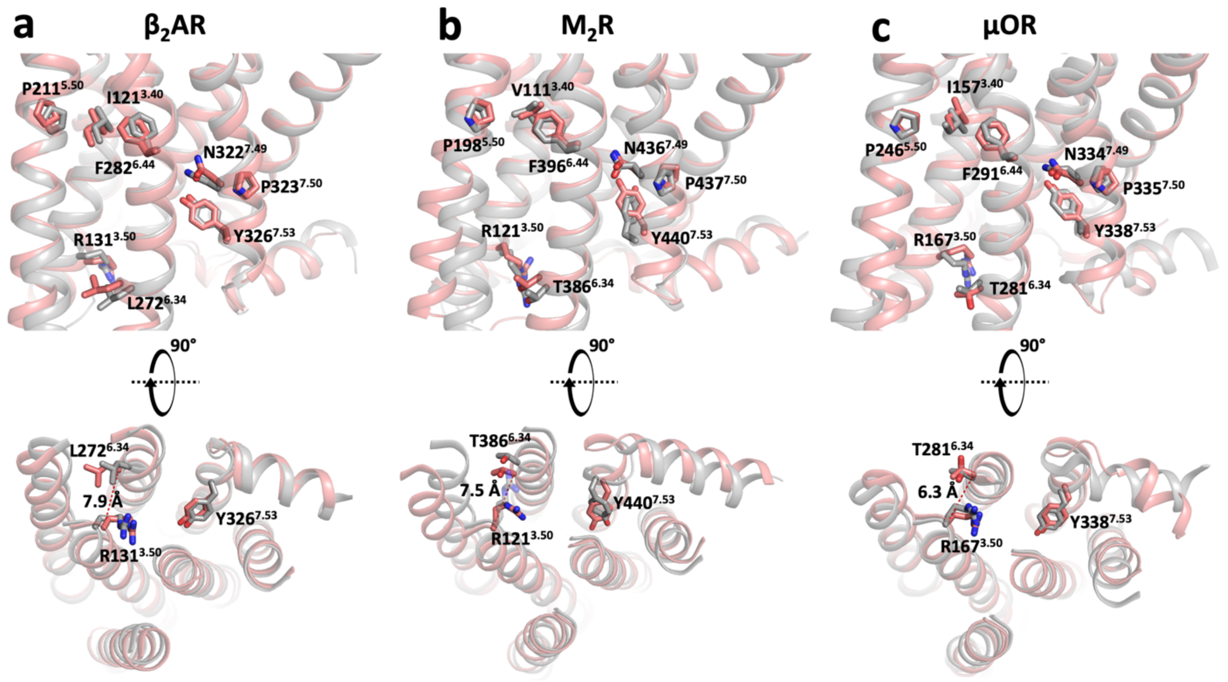

Supplementary Fig. 10: Comparison of calculated inactive states with corresponding inactive-state crystal structures of β2AR (a), M2R (b) and μOR (c). Calculated inactive models are shown in red and inactive-state X-ray crystal structures in gray (β2AR: PDB-ID 2RH1; M2R: PDB 5ZKC; μOR: PDB 4DKL). Key residues comprising the $P^{5.50}$-$I^{3.40}$-$F^{6.44}$/ $P^{5.50}$-$V^{3.40}$-$F^{6.44}$ and the $N^{7.49}P^{7.50}xxY^{7.53}$ motif as well as residues in positions 3.50 and 6.34 are shown in sticks. Calculated RMSDs between the inactive-state model and the inactive X-ray crystal structures of β2AR: 1.9 Å (transmembrane regions), 1.7 Å (TM 6), 1.0 Å ($N^{7.49}P^{7.50}xxY^{7.53}$), 1.3 Å ($P^{5.50}$-$I^{3.40}$-$F^{6.44}$); M2R: 2.2 Å (transmembrane regions), 1.8 Å (TM 6), 1.4 Å ($N^{7.49}P^{7.50}xxY^{7.53}$), 0.6 Å ($P^{5.50}$-$V^{3.40}$-$F^{6.44}$) and μOR: 2.2 Å (transmembrane regions), 1.0 Å (TM 6), 1.1 Å ($N^{7.49}P^{7.50}xxY^{7.53}$), 1.1 Å ($P^{5.50}$-$I^{3.40}$-$F^{6.44}$). Source data are provided as a Source Data file.

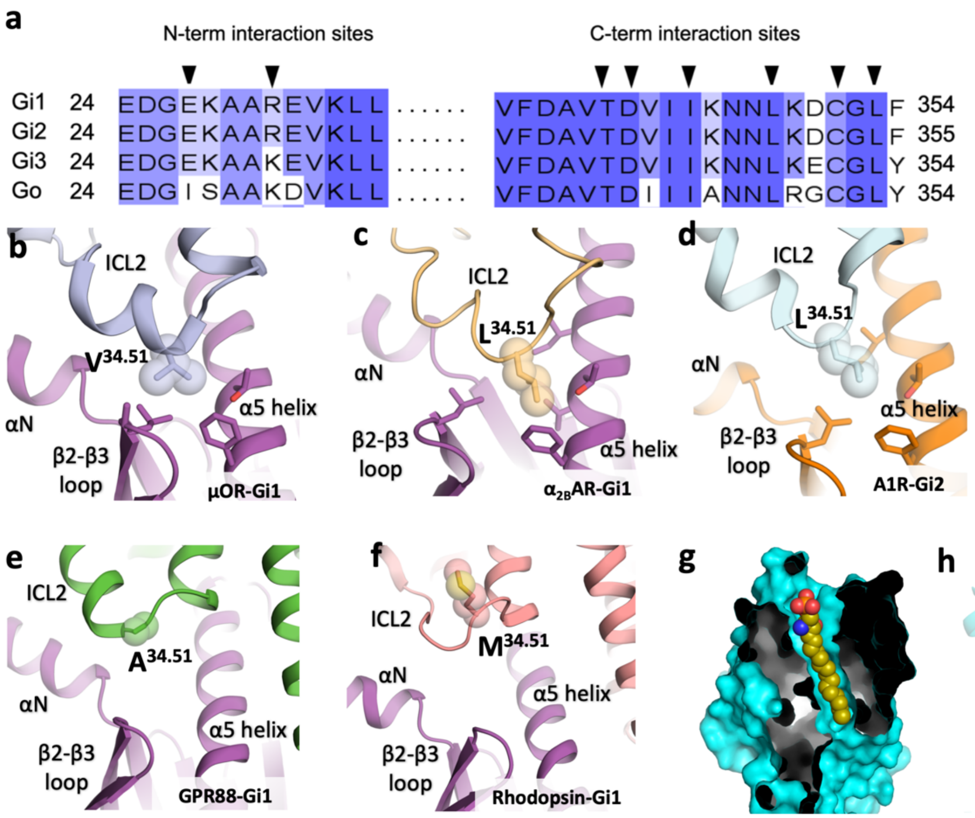

Supplementary Fig. 11: Comparison of interactions between ICL2 and G-protein and the S1P binding pocket a Sequence alignment of the N-terminus and C-terminus of different Gi/o family G-proteins. **b-d** Hydrophobic interactions between residue 34.51 and the pocket form by αN, β2-β3 loop and α5 of Gα were observed for μOR-Gi1 (**b,** PDB: 6DDE), α_{2B}AR-Gi1 (**c,** PDB: 6K42) and A1R-Gi2 (**d,** PDB: 6D9H). **e,f** Residue 34.51 positioned away from the Gα hydrophobic pocket for GPR88-Gi1 (**e**) and rhodopsin-Gi1(**f,** PDB: 6CMO). **g,h** The orthosteric binding pocket of S1P in S1PR3 (PDB: 7C4S). A hydrophobic tunnel is formed by TM3, TM4 and TM5, similar to the 2-PCCA orthosteric pocket in GPR88.

Supplementary Table 1. Cryo-EM data collection, refinement and validation statistics.

Data collection and processing	2-PCCA-GPR88-Gi1<br>EMD-31164 PDB ID 7EJX	GPR88-Gi1<br>EMD-32904 PDB ID 7WZ4
Magnification	105,000	105,000
Voltage (kV)	300	300
Electron exposure (e^-/Å)	53	53
Defocus range (μm)	-1.0 to -2.0	-1.0 to -2.0
Pixel size (Å)	0.83	0.85
Symmetry imposed	C1	C1
Initial particle images (no.)	4647118	3166931
Final particle images (no.)	988958	314834
Map resolution (Å)	2.4	3.0
FSC threshold	0.143	0.143
**Refinement**		
Initial model used (PDB code)	6DDE	7EJX
Map sharpening B-factor (Å)	-80	-80
**Model composition**		
Non-hydrogen atoms	8309	8187
Protein residues	1073	1075
**B factor (Å)**		
Protein	76.38	76.77
Ligand	99.22	
**R.m.s. deviations**		
Bond lengths (Å)	0.004	0.003
Bond angles (°)	0.955	0.580
**Validation**		
MolProbity score	1.63	1.24
Clashscore	10	3.2
**Ramachandran plot**		
Favored (%)	97.23	97.34
Allowed (%)	2.77	2.66
Disallowed (%)	0	0
