# Supplementary material for: Activation and allosteric regulation of the orphan GPR88-Gi1 signaling complex
Source: Nat Commun. 2022 May 2;13:2375. doi: 10.1038/s41467-022-30081-5 (PMC9061749; doi:10.1038/s41467-022-30081-5)
Supplement: Supplementary file 3 — Description of Additional Supplementary Files [file 41467_2022_30081_MOESM3_ESM.pdf]

Supplementary Data 1.

The PDB file of the calculated inactive GPR88 model
