# Supplementary material for: Activation and allosteric regulation of the orphan GPR88-Gi1 signaling complex
Source: Nat Commun. 2022 May 2;13:2375. doi: 10.1038/s41467-022-30081-5 (PMC9061749; doi:10.1038/s41467-022-30081-5)
Supplement: Supplementary file 5 — Reporting Summary [file 41467_2022_30081_MOESM5_ESM.pdf]

nature portfolio

Corresponding author(s): Yang Du

Last updated by author(s): Mar 21, 2022

Reporting Summary

Nature Portfolio wishes to improve the reproducibility of the work that we publish. This form provides structure for consistency and transparency in reporting. For further information on Nature Portfolio policies, see our Editorial Policies and the Editorial Policy Checklist.

Statistics

For all statistical analyses, confirm that the following items are present in the figure legend, table legend, main text, or Methods section.

n/a	Confirmed	
☐	☒	The exact sample size (*n*) for each experimental group/condition, given as a discrete number and unit of measurement
☐	☒	A statement on whether measurements were taken from distinct samples or whether the same sample was measured repeatedly
☐	☒	The statistical test(s) used AND whether they are one- or two-sided<br>*Only common tests should be described solely by name; describe more complex techniques in the Methods section.*
☒	☐	A description of all covariates tested
☐	☒	A description of any assumptions or corrections, such as tests of normality and adjustment for multiple comparisons
☐	☒	A full description of the statistical parameters including central tendency (e.g. means) or other basic estimates (e.g. regression coefficient) AND variation (e.g. standard deviation) or associated estimates of uncertainty (e.g. confidence intervals)
☐	☒	For null hypothesis testing, the test statistic (e.g. *F*, *t*, *r*) with confidence intervals, effect sizes, degrees of freedom and *P* value noted<br>*Give P values as exact values whenever suitable.*
☒	☐	For Bayesian analysis, information on the choice of priors and Markov chain Monte Carlo settings
☒	☐	For hierarchical and complex designs, identification of the appropriate level for tests and full reporting of outcomes
☒	☐	Estimates of effect sizes (e.g. Cohen's *d*, Pearson's *r*), indicating how they were calculated

Our web collection on statistics for biologists contains articles on many of the points above.

Software and code

Policy information about availability of computer code

Data collection	SoftMax Pro 7.0.3, the movies stacks was automatically collected using the SerialEM 3.7, MODELLER version 9.4 for modeling of missing protein loops, UCSF Chimera version 1.12 for preparation of the receptor, AMBER Tools version 18 # for assignment of ligand parameters + coordinate/topology file generation, GROMACS 2018.4 patched with PLUMED 2.5.0 for performing metadynamics simulations.
Data analysis	GraphPad Prism 7 and 8, FlowJo 10.7, CryoSPARC 3.2.1, Relion 3.0, MotionCor2 1.4.0, GCTF 1.18, JSPR, PYEM 2.0.0, UCSF Chimera 1.12, Coot 0.9.2, Phenix 1.18.2, MolProbity 4.2, PyMOL 2.1, Molaris-XG 9.15 and PLUMED version 2.5.0 for reconstruction of the FES and error estimation (using block analysis according to the "Master ISDD tutorial 2020: Metadynamics simulations with PLUMED"), GROMACS 2018.4 patched with PLUMED 2.5.0 for analysis of the trajectories, Matplotlib version 3.4.3 # for creating the FES plots including error estimates.

For manuscripts utilizing custom algorithms or software that are central to the research but not yet described in published literature, software must be made available to editors and reviewers. We strongly encourage code deposition in a community repository (e.g. GitHub). See the Nature Portfolio guidelines for submitting code & software for further information.

Data

Policy information about availability of data

All manuscripts must include a data availability statement. This statement should provide the following information, where applicable:

- Accession codes, unique identifiers, or web links for publicly available datasets
- A description of any restrictions on data availability
- For clinical datasets or third party data, please ensure that the statement adheres to our policy

All data generated or analyzed during this study are included in this article and its Supplementary Information. Calculated inactive receptor models (obtained by metadynamics simulations) are provided as supplementary PDB files. The 3D Cryo-EM density maps of the 2-PCCA-bound and apo GPR88-Gi-scFv16 complex have

been deposited in the Electron Microscopy Data Bank database under accession codes EMD-31164 and EMD-32904, respectively. The atomic coordinates for the atomic models of the 2-PCCA-GPR88-Gi-scFv16 complexes generated in this study have been deposited in the Protein Data Bank database under accession codes 7EJX and 7WZ4, respectively. The raw data for the main Fig. 1b, 1c, 2h-j, 4 and Supplementary Fig. 1b, 1c, 4, 5e, 7, 8b-e, 10 generated in this study are provided in the Source Data file. The structural models of rhodopsin-Gi1, μOR-Gi1, α2BAR-Gi1 and A1R-Gi2 used in this study are available in the Protein Data Bank database under accession codes 6CMO, 6DDE, 6K42 and 6D9H, respectively. Source data are provided with this paper.

Field-specific reporting

Please select the one below that is the best fit for your research. If you are not sure, read the appropriate sections before making your selection.

☒ Life sciences ☐ Behavioural & social sciences ☐ Ecological, evolutionary & environmental sciences

For a reference copy of the document with all sections, see nature.com/documents/nr-reporting-summary-flat.pdf

Life sciences study design

All studies must disclose on these points even when the disclosure is negative.

Sample size	No statistical method was used to determine the sample size. We repeated each in vitro experiment at least three times and results were reproducibly obtained.
Data exclusions	No data were systematically excluded.
Replication	In vitro experiments were independently performed at least three times. All attempts at replication were succeeded.
Randomization	No randomization was attempted or needed." This was not a clinical trial or animal study that is dependent on randomization.
Blinding	No blinding was attempted or needed. There was no group allocation performed in this study.

Reporting for specific materials, systems and methods

We require information from authors about some types of materials, experimental systems and methods used in many studies. Here, indicate whether each material, system or method listed is relevant to your study. If you are not sure if a list item applies to your research, read the appropriate section before selecting a response.

Materials & experimental systems

n/a	Involved in the study	
☐	☒	Antibodies
☐	☒	Eukaryotic cell lines
☒	☐	Palaeontology and archaeology
☒	☐	Animals and other organisms
☒	☐	Human research participants
☒	☐	Clinical data
☒	☐	Dual use research of concern

Methods

n/a	Involved in the study	
☒	☐	ChIP-seq
☐	☒	Flow cytometry
☒	☐	MRI-based neuroimaging

Antibodies

Antibodies used	anti-DYKDDDDK mouse monoclonal antibody (Wako Pure Chemicals, Clone 1E6, catalog no. 018-22381, https://labchem-wako.fujifilm.com/jp/product/detail/W01W0101-2238.html) and goat anti-mouse IgG secondary antibody conjugated with Alexa Fluor 488 (Thermo Fisher Scientific, catalog no. A32723, https://www.thermofisher.com/antibody/product/Goat-anti-Mouse-IgG-H-L-Highly-Cross-Adsorbed-Secondary-Antibody-Polyclonal/A32723); scFV16 was purified to be used for the GPR88-Gi complex assembly.
Validation	The purchased antibodies were well validated by the manufactures in their specific data sheets.

Eukaryotic cell lines

Policy information about cell lines

Cell line source(s)	HEK293A cells (Thermo Fisher Scientific, Cat R70507); Sf9 cells (Expresssion Systems, Cat 94-001F); Tni cells (Expression Systems, Cat 94-002F)
Authentication	Cell lines are maintained by the supplier. No additional authentication was performed by the authors of this study.
Mycoplasma contamination	HEK293A cells have been screened to ensure the absence of mycoplasma contamination using MycoAlert Mycoplasma

Mycoplasma contamination	detection kit (Lonza).
Commonly misidentified lines (See ICLAC register)	None used.

Flow Cytometry

Plots

Confirm that:

☐ The axis labels state the marker and fluorochrome used (e.g. CD4-FITC).

☐ The axis scales are clearly visible. Include numbers along axes only for bottom left plot of group (a 'group' is an analysis of identical markers).

☐ All plots are contour plots with outliers or pseudocolor plots.

☐ A numerical value for number of cells or percentage (with statistics) is provided.

Methodology

Sample preparation	HEK293 cells transiently expressing a test FLAG-GPR88 construct along with the NanoBiT-Gi1 sensor were labeled with the anti-DYKDDDDK tag, followed by the Alexa Fluor 488-conjugated anti-mouse IgG secondary antibody.
Instrument	EC800 flow cytometer (Sony)
Software	FlowJo software (FlowJo)
Cell population abundance	N/A. Cell population abundance analysis is not needed in this study.
Gating strategy	N/A. We used all of the recorded fluorescent signals and calculated a simple mean value. Therefore, description of gating strategy or the plot representation was not performed.

☒ Tick this box to confirm that a figure exemplifying the gating strategy is provided in the Supplementary Information.
